# Skeletal muscle energy metabolism in environmental hypoxia: climbing towards consensus

**DOI:** 10.1186/2046-7648-3-19

**Published:** 2014-11-28

**Authors:** James A Horscroft, Andrew J Murray

**Affiliations:** Department of Physiology, Development and Neuroscience, University of Cambridge, Downing Street, CB2 3EG Cambridge, UK

**Keywords:** Hypoxia, High altitude, Skeletal muscle, Mitochondria, Metabolism

## Abstract

Skeletal muscle undergoes metabolic remodelling in response to environmental hypoxia, yet aspects of this process remain controversial. Broadly, environmental hypoxia has been suggested to induce: (i) a loss of mitochondrial density; (ii) a substrate switch away from fatty acids and towards other substrates such as glucose, amino acids and ketone bodies; and (iii) a shift from aerobic to anaerobic metabolism. There remains a lack of a consensus in these areas, most likely as a consequence of the variations in degree and duration of hypoxic exposure, as well as the broad range of experimental parameters used as markers of metabolic processes. To attempt to resolve some of the controversies, we performed a comprehensive review of the literature pertaining to hypoxia-induced changes in skeletal muscle energy metabolism. We found evidence that mass-specific mitochondrial function is decreased prior to mass-specific mitochondrial density, implicating intra-mitochondrial changes in the response to environmental hypoxia. This loss of oxidative capacity does not appear to be matched by a loss of glycolytic capacity, which on the whole is not altered by environmental hypoxia. Environmental hypoxia does however induce a selective attenuation of fatty acid oxidation, whilst glucose uptake is maintained or increased, perhaps to support glycolysis in the face of a downregulation of oxidative metabolism, optimising the pathways of ATP synthesis for the hypoxic environment.

**Electronic supplementary material:**

The online version of this article (doi:10.1186/2046-7648-3-19) contains supplementary material, which is available to authorized users.

## Review

### Background

**Figure 1:**
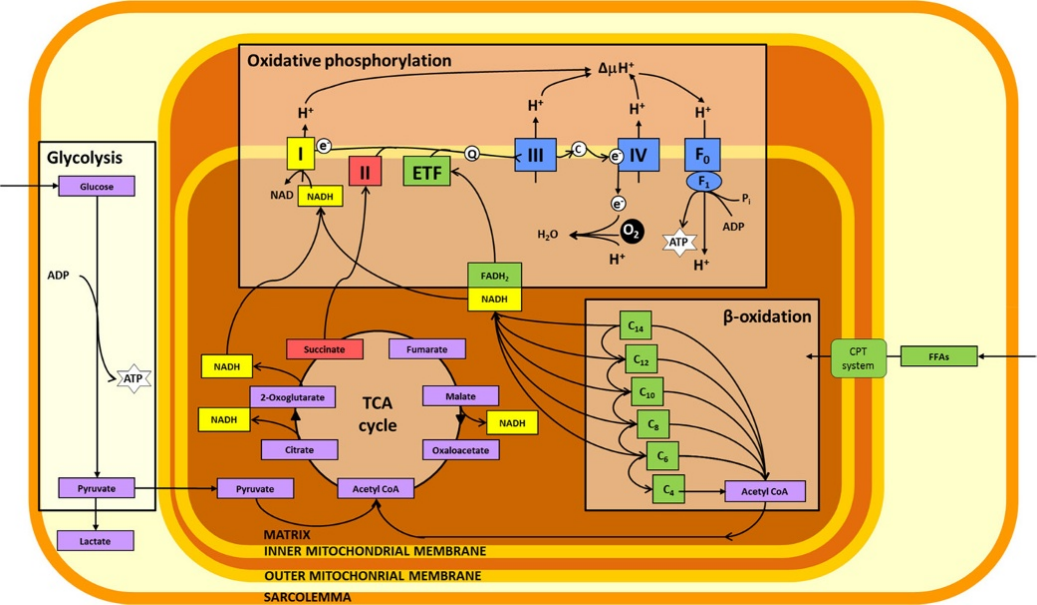
**Energy metabolism in the skeletal muscle.** Glycolysis represents an oxygen-independent source of ATP and pyruvate. Pyruvate is reduced in the cytosol to form lactate or oxidised in the mitochondrial matrix to form acetyl CoA, which feeds into the TCA cycle. β-oxidation of fatty acids and the TCA cycle produce reduced intermediates, NADH and FADH_2_, which are oxidised by complexes of the electron transport chain. Electrons are transferred to the final oxygen acceptor, O_2_, and the free energy from this process is used to pump H^+^ ions into the intermembrane space. The resulting electrochemical gradient is the driving force for the oxidative phosphorylation of ADP. *ETF* electron-transferring flavoprotein, *I-IV* complexes of the electron transport chain, *F*_*0*_ and *F*_*1*_ subunits of the ATP synthase, *NADH* β-nicotinamide adenine dinucleotide reduced, *NAD* β-nicotinamide adenine dinucleotide, *C*_*n*_ acetyl CoA with carbon chain length *n*, *FFA* free fatty acids. Figure adapted from [2].

Skeletal muscle, like all oxidative tissues of the body, is critically dependent on a supply of oxygen to maintain energetic and redox homeostasis. ATP can be synthesised in the skeletal muscle in an oxygen-dependent manner in the mitochondria via oxidative phosphorylation, utilising substrates such as glycolytically derived pyruvate, fatty acids, amino acids and ketone bodies, but also in an oxygen-independent manner in the cytosol, via glycolysis with the resulting pyruvate converted to lactate (Figure 1). Under conditions of a plentiful oxygen supply, however, oxidative phosphorylation would normally meet the majority of the cell’s ATP requirements [1], due to the greater range of substrates available and the much higher yield of ATP derived from glucose.

Environmental hypoxia, either in a hypobaric/normobaric hypoxia chamber or at high altitude, decreases the partial pressure of arterial oxygen (Pa(O_2_)). In order to compensate for this, oxygen delivery is improved via changes in resting ventilation rate, circulating haemoglobin concentration and capillary density [3], whilst metabolic remodelling at the tissues might alter oxygen utilisation. Studies in cultured cells suggest that the transcription factor, hypoxia-inducible factor 1-alpha (HIF1α), is upregulated in hypoxia, increasing glycolysis [4] and thereby attenuating oxygen utilisation and ATP synthesis [5]. A loss of cellular mitochondrial content may be driven by the downregulation of mitochondrial biogenesis factors such as peroxisome proliferator-activated receptor γ co-activator 1 alpha or beta (PGC1α/β) in tandem with the upregulation of mitochondrial autophagy factors such as BCL2/adenovirus E1B 19 kDa interacting protein (BNIP3) [6]. Meanwhile, the upregulation of pyruvate dehydrogenase kinase (PDK) isoforms deactivates pyruvate dehydrogenase, which impairs pyruvate entry into the TCA cycle, resulting in a high rate of glycolysis relative to oxidative phosphorylation, the Warburg effect [7, 8]. Finally, the efficiency of mitochondrial electron transfer and thus oxygen utilisation is improved by a HIF1α-dependent switch in subunits at complex IV [9].

Despite this valuable mechanistic work in cell cultures, there remains a paucity of research into the effects of environmental hypoxia on energy metabolism in different mammalian tissues *in vivo*. The skeletal muscle is an interesting model tissue, as it has a relatively high capacity for respiration, with metabolic rates altered acutely by exertion and numerous metabolic features (for example, mitochondrial density and/or substrate preference) altered chronically by, e.g. training [10], diet [10] and environmental factors [11]. In humans, the muscle is easily accessible for biopsy, even under field conditions.

The aim of this review was to collate evidence pertaining to the remodelling of metabolic processes in mammalian skeletal muscle *in vivo* in response to environmental hypoxia, accounting for variations in degree and duration of hypoxic exposure.

### Methods

#### Search strategy

A search protocol was developed to identify relevant research articles with unbiased results. The search term ‘(altitude OR hypoxia) AND “skeletal muscle” AND (mitochondria OR glycolysis OR “fatty acid” OR “oxidative phosphorylation”)’ was entered into the database PubMed in June 2014, and the titles and abstracts of all results were assessed for relevance. The reference lists of review articles arising from this initial search were reviewed for research papers which did not appear in the original search, and any relevant articles were also included. Any publication date or animal model was accepted for inclusion, providing that a skeletal muscle was studied. Finally, any type (e.g. ascent to altitude, habitation of a hypoxic chamber, ischaemia and anaemia), intensity, duration and frequency of hypoxic exposure was considered acceptable for more thorough analysis.

#### Search results

**Figure 2:**
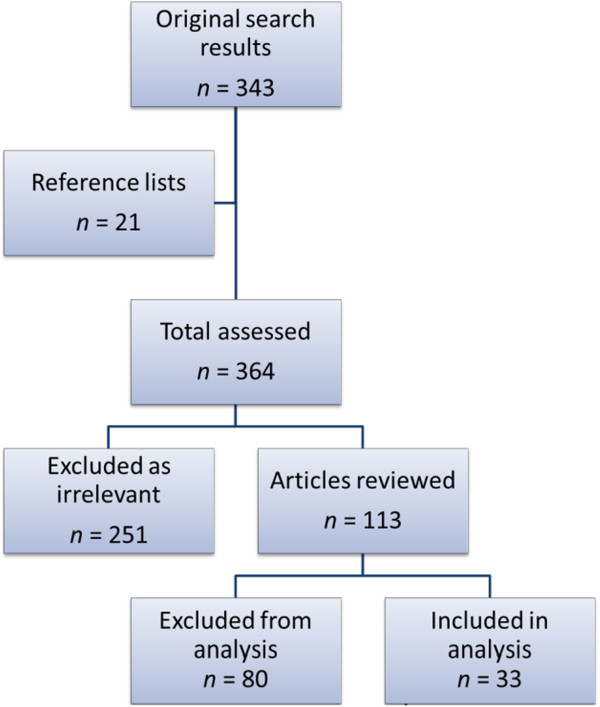
**Selection process for identifying relevant papers in the literature.**

The search returned 343 results in June 2014. A further 21 papers cited in reviews found by the initial search term were added due to relevance. Of these 364 papers, 251 were excluded as irrelevant and 113 reviewed in detail. An aim of this review was to investigate the consequences of variations in degree and duration of hypoxic exposure on mammalian muscle energy metabolism. Thus, from the articles identified as relevant, we selected those in which a mammal was exposed to continuous environmental hypoxia of greater than 1 day and aspects of skeletal muscle energy metabolism were assessed. Where possible, observations that may have been influenced by confounding factors were excluded. To this end, studies using genetically manipulated animal models, pre-acclimatised or evolutionarily adapted human cohorts, or confounding interventions such as exercise or pharmacological agents, were excluded. This left 33 articles, of which 14 used human *m. vastus lateralis*, 6 used a mouse skeletal muscle and 13 used a rat skeletal muscle. A flowchart of the selection process is shown in Figure 2, and further details of the reasons for exclusion are given in Additional file 1: Table S1.

#### Data extraction

In the remaining 33 articles, we recorded all reported observations that could be used as a marker of one of four metabolic processes of interest (glycolysis, β-oxidation, TCA cycle and oxidative phosphorylation) plus mitochondrial density. Ketolysis, amino acid metabolism and high-energy phosphate transfer were excluded, as there were very few observations of biomarkers of these processes. Expression, levels or activity of appropriate enzymes; expression and levels of regulating transcription factors; and functional respirometry data were considered as markers (Table 1).

**Table 1:** **Accepted biomarkers for glycolysis, β-oxidation, TCA cycle function, oxidative phosphorylation and mitochondrial density**

Aspect of metabolism	Biomarkers			
	Expression, levels or activity of enzymes/transporters	Expression, levels or activity of regulators	Rate measurements	Other validated markers [ [13]]
**Glycolysis**				
	Monocarboxylate transporters (MCT)			
	Hexokinase (HK)			
	Phosphoglucose isomerase (PGI)			
	Phosphofructokinase (PFK)			
	Aldolase (ALD)			
	Triose phosphate isomerase (TPI)			
	Glyceraldehyde 3-phosphate dehydrogenase (G3PDH)			
	Phosphoglycerate kinase (PGK)			
	Phosphoglycerate mutase (PGM)			
	Enolase (ENO)			
	Pyruvate kinase (PyK)			
	Lactate dehydrogenase (LDH)			
			Glucose oxidation	
**β-oxidation**				
	Carnitine acylcarnitine translocase (CACT)			
	Carnitine palmitoyl transferases (CPT)			
	Acyl CoA dehydrogenases			
	Enoyl CoA hydratase (ECAH)			
	Enoyl CoA isomerase (ECAI)			
	L-3-hydroxyacyl CoA dehydrogenase (HOAD)			
	Thiolase (THI)			
		PPARα		
			Uptake/utilisation of fatty acids	
			Oxidative phosphorylation with fatty acid substrates	
**TCA cycle**				
	Pyruvate dehydrogenase			
	Citrate synthase			
	Aconitase			
	Isocitrate dehydrogenase			
	α-ketoglutarate dehydrogenase			
	Succinyl CoA synthetase			
	Succinate dehydrogenase			
	Fumarase			
	Malate dehydrogenase			
**Oxidative phosphorylation**				
	Complex I			
	Complex II			
	Complex III			
	Complex IV			
	Complex V			
	Electron transferring flavoprotein (ETF)			
			Oxidative phosphorylation	
**Mitochondrial density (mitochondrial density measurements by electron microscopy)**				
	Bax			
	Bcl-2*			
	BNIP3*			
	PGC-1α			
				Citrate synthase activity
				Complex IV activity

*biomarkers used as negative indicators of the process.

#### Data analysis

The degree and duration of hypoxic exposure was noted and has been described uniformly in this review. Degree is reported as an estimate of the minimum atmospheric partial pressure of oxygen p(O_2_)_min_ reached by every member of the cohort during each study. Duration is reported as the total time spent in an environment with a p(O_2_) <15.0 kPa (equivalent to being >3,000 m above sea level). Where hypoxic degree was not reported in p(O_2_), conversions were made to estimate the p(O_2_)_min_ in the reported condition using the following formula, adapted from West 1996 [12] where *h* is the height above sea level in kilometres.


If appropriate, the results reported in each paper were sub-divided into those pertaining to different experimental “settings”. We define a setting as a uniform hypoxic challenge (degree and duration), exerted on one particular species and muscle or muscle group within a single study.

For each setting, all biomarkers described in Table 1 were considered and are reported here. In addition, a single result for each of the four metabolic processes and mitochondrial density was inferred from each setting as follows: *increase* (where at least one biomarker of a process was significantly increased by hypoxia, and none decreased); *decrease* (where at least one biomarker of a process was significantly decreased by hypoxia, and none increased); *unchanged* (where at least one biomarker was measured and no biomarkers were significantly altered by hypoxia); and *unclear* (where at least one biomarker of a process was significantly increased and another significantly decreased). In the case of a conflict in results, however, where a direct measurement was taken (e.g. mitochondrial density by electron microscopy), this was given priority over an established indirect proxy (e.g. mitochondrial density by citrate synthase activity) [13], which in turn was given priority over expression, levels or activity of known regulators of that process (e.g. PGC1α). This occurred in one instance in the study by Chaillou *et al.* [14], where two established markers of mitochondrial density (citrate synthase activity and complex IV activity) decreased in a rat *plantaris* muscle, whilst one upstream regulator of mitochondrial biogenesis (PGC1α) increased. This setting was thus labelled as a *decrease*.

To untangle the effects of different degrees and durations of hypoxia, observations were sub-categorised by severity in terms of atmospheric partial pressure of O_2_ (p(O_2_)): high (11.7 < p(O_2_) ≤15.0 kPa, ca. 3,000–5,000 m above sea level), very high (10.0 < p(O_2_) ≤11.7 kPa, ca. 5,000–6,250 m above sea level) or extreme (p(O_2_) ≤10.0 kPa, ca. 6,250+ m above sea level); and duration (*t*): short term (0 < *t* ≤14 d in hypoxia), medium term (14 < *t* ≤ 42 d) and long term (t > 42 *d*).

### Results

#### Glycolysis

For biomarkers of glycolysis, 25 hypoxic settings were identified across 15 papers, the results of which are summarised in Table 2. The markers of glycolysis in human *m. vastus lateralis* decreased in four settings [15–18], increased in two [19, 20], remained unchanged in five [18, 20–22] and were unclear in one [15]. Similar patterns were found in rodents [23–28] and appeared to be unrelated to the degree of hypoxic exposure. The effect of hypoxia on individual glycolytic enzymes does not reveal a striking pattern, with most unchanged, significantly increased or significantly decreased in one of the studies.

**Table 2:** **The effects of environmental hypoxia on biomarkers of glycolysis in skeletal muscle**

***First author***	***Year***	***Organism***	***Muscle model***	***Hypoxia model***	***p(O***_***2***_ ***)***_***min***_ ***(kPa)***	***Duration (d)***	***Marker***	***Change***
Green [15]	1992	Human	vl	4,300 m	12.8	1	Phosphofructokinase activity	**↓**
							Hexokinase activity	**=**
Roberts [20]	1996	Human	vl	4,300 m	12.8	1	Glucose oxidation	**=**
Pastoris [29]	1995	Rat	gnm	10% O_2_	10.1	3	Hexokinase activity	**=**
							Phosphofructokinase activity	**↑**
							Lactate dehydrogenase activity	**=**
							Pyruvate kinase activity	**↓**
Pastoris [29]	1995	Rat	sol	10% O_2_	10.1	3	Hexokinase activity	**=**
							Phosphofructokinase activity	**↓**
							Lactate dehydrogenase activity	**=**
							Pyruvate kinase activity	**=**
Dutta [28]	2009	Rat	mix	349 mmHg	10.3	7	Lactate dehydrogenase activity	**↑**
Vigano [16]	2008	Human	vl	4,559 m	12.4	8	Enolase levels	**↓**
van Hall [21]	2009	Human	vl	4,100 m	13.1	14	Lactate dehydrogenase activity	**=**
De Palma [27]	2007	Rat	gnm	10% O_2_	10.1	14	β-enolase levels	**↓**
							Phosphoglyercomutase 2 levels	**↓**
							Pyruvate kinase levels	**↑**
							Triose phosphate isomerase levels	**↓**
Young [22]	1984	Human	vl	4,300 m	12.8	18	Hexokinase activity	**=**
							Lactate dehydrogenase activity	**=**
Levett [18]	2012	Human	vl	5,300 m	11.3	19	Hexokinase activity	**=**
Roberts [30]	1996	Human	vl	4,300 m	12.8	21	Glucose oxidation	**↑**
Green [15]	1992	Human	vl	4,300 m	12.8	21	Phosphofructokinase activity	**↓**
							Hexokinase activity	**↑**
Daneshrad [24]	2000	Rat	sol	10% O_2_	10.1	21	Hexokinase activity	**↑**
							Lactate dehydrogenase activity	**=**
							Phosphofructokinase activity	**=**
							Pyruvate kinase levels	**=**
Green [19]	2000	Human	vl	6,194 m	10.1	21	Lactate dehydrogenase activity	**↑**
Green [17]	1989	Human	vl	8,848 m	7.1	40	Hexokinase activity	**↓**
							α-GPDH activity	**=**
							Lactate dehydrogenase activity	**=**
							Phosphofructokinase activity	**=**
							Pyruvate kinase levels	**=**
van Hall [21]	2009	Human	vl	4,100 m	13.1	56	Lactate dehydrogenase activity	**=**
McClelland [25]	2002	Rat	sol	4,300 m	12.8	56	Lactate dehydrogenase levels	**=**
							Monocarboxylate transporter 1 levels	**=**
							Monocarboxylate transporter 4 levels	**↓**
McClelland [25]	2002	Rat	pla	4,300 m	12.8	56	Lactate dehydrogenase levels	**=**
							Monocarboxylate transporter 1 levels	**↓**
							Monocarboxylate transporter 4 levels	**↓**
McClelland [25]	2002	Rat	gnm	4,300 m	12.8	56	Lactate dehydrogenase levels	**=**
							Monocarboxylate transporter 1 levels	**=**
							Monocarboxylate transporter 4 levels	**=**
Abdelmalki [23]	1996	Rat	sol	13% O_2_	13.1	64	Lactate dehydrogenase activity	**=**
							Phosphofructokinase activity	**↑**
							Hexokinase activity	**=**
Abdelmalki [23]	1996	Rat	pla	13% O_2_	13.1	64	Lactate dehydrogenase activity	**=**
							Phosphofructokinase activity	**=**
							Hexokinase activity	**=**
Abdelmalki [23]	1996	Rat	rq	13% O_2_	13.1	64	Lactate dehydrogenase activity	**=**
							Phosphofructokinase activity	**=**
Abdelmalki [23]	1996	Rat	wq	13% O_2_	13.1	64	Lactate dehydrogenase activity	**=**
							Phosphofructokinase activity	**↑**
Levett [18]	2012	Human	vl	8,848 m	7.1	66	Hexokinase activity	**↓**
Ou [26]	2004	Rat	edl	5,500 m	11.0	90	Lactate dehydrogenase activity	**=**

↑ Change in biomarker is indicative of an *increase* in β-oxidation in hypoxia.

= No change in biomarker in hypoxia.

↓ Change in biomarker is indicative of a *decrease* in β-oxidation in hypoxia.

*Abbreviations:* *edl extensor digitorum longus*, *mix* mixed skeletal, *pla plantaris*, *q quadriceps*, *rq* red *quadriceps*, sol *soleus*, vl *vastus lateralis*, wq white *quadriceps*.

#### β-oxidation

For biomarkers of β-oxidation, 22 hypoxic settings were identified across 15 papers, the results of which are summarised in Table 3. There was a tendency towards a decrease in β-oxidation following a hypoxic stimulus, with a decrease in at least one biomarker reported in 8/22 settings [16, 18, 23, 28, 30–32] and none showing an increase. A commonly used marker of β-oxidation was the activity of 3-hydroxyacyl-CoA dehydrogenase (HOAD). HOAD activity was unchanged in five settings [15, 17, 18, 33] and decreased in one setting [18] in humans, with a similar ratio of results in rodents [23, 24, 28, 31, 32, 34]. Assessment of levels and/or activity of proteins associated with mitochondrial fatty acid import, e.g. carnitine-acylcarnitine translocase (CACT) [16] and carnitine pamitoyltransferase 1 (CPT1) [32] suggested that these are decreased by sustained hypoxia, an effect possibly mediated through the HIF-PPARα signalling axis, as levels of peroxisome proliferator-activated receptor alpha (PPARα) were lowered by environmental hypoxia in mice [31]. Acyl-carnitine-supported respirometry rates were lower following hypoxic exposure, when malate plus palmitoyl carnitine [31, 32], but not octanoyl carnitine [35, 36], were used as substrates.

**Table 3:** **The effects of environmental hypoxia on biomarkers of β-oxidation in skeletal muscle**

***First author***	***Year***	***Organism***	***Muscle model***	***Hypoxia model***	***p(O***_***2***_ ***)***_***min***_ ***(kPa)***	***Duration (d)***	***Marker***	***Change***
Green [15]	1992	Human	vl	4,300 m	12.8	1	HOAD activity	**=**
Roberts [30]	1996	Human	vl	4,300 m	12.8	1	Fatty acid oxidation	**=**
Morash [31]	2013	Mouse	mix	13% O_2_	13.1	1	PPARα levels	**↓**
							CPT-1 levels	**↓**
							CPT-1 activity	**↓**
							HOAD activity	**=**
							Palmitate oxidation	**↓**
							Palmitoyl carnitine OXPHOS	**↓**
Dutta [28]	2009	Rat	mix	349 mmHg	10.3	7	CPT-1 activity	**=**
							Fatty acid oxidation	**↓**
							HOAD activity	**↓**
Morash [31]	2013	Mouse	mix	13% O_2_	13.1	7	PPARα levels	**↓**
							CPT-1 levels	**↓**
							CPT-1 activity	**↓**
							HOAD activity	**=**
							Palmitate oxidation	**↓**
							Palmitoyl carnitine OXPHOS	**↓**
Vigano [16]	2008	Human	vl	4,559 m	12.4	8	CACT levels	**↓**
							ECAH levels	**↓**
							ECAI levels	**↓**
Jacobs [35]	2013a	Human	vl	4,559 m	12.4	10	Octanoyl carnitine OXPHOS	**=**
Levett [18]	2012	Human	vl	5,300 m	11.3	19	HOAD activity	**=**
Daneshrad [24]	2000	Rat	sol	10% O_2_	10.1	21	HOAD activity	**=**
Green [15]	1992	Human	vl	4,300 m	12.8	21	HOAD activity	**=**
Roberts [30]	1996	Human	vl	4,300 m	12.8	21	Fatty acid oxidation	**↓**
Takahashi [34]	1993	Rat	pla	10% O_2_	10.1	28	HOAD activity	**=**
Takahashi [34]	1993	Rat	sol	10% O_2_	10.1	28	HOAD activity	**=**
Jacobs [36]	2013b	Human	vl	3,454 m	14.2	28	Octanoyl carnitine OXPHOS	=
Galbes [32]	2008	Rat	q	4,000 m	13.3	35	CPT-1 activity	**↓**
							CPT-1 levels	**↓**
							HOAD activity	**↓**
							Palmitoyl carnitine OXPHOS	**↓**
Green [17]	1989	Human	vl	8,848 m	7.1	40	HOAD activity	**=**
Abdelmalki [23]	1996	Rat	sol	13% O_2_	13.1	64	HOAD activity	**=**
Abdelmalki [23]	1996	Rat	pla	13% O_2_	13.1	64	HOAD activity	**↓**
Abdelmalki [23]	1996	Rat	rq	13% O_2_	13.1	64	HOAD activity	**=**
Abdelmalki [23]	1996	Rat	wq	13% O_2_	13.1	64	HOAD activity	**=**
Levett [18]	2012	Human	vl	8,848 m	7.1	66	HOAD activity	**↓**
Mizuno [33]	2008	Human	vl	5,250 m	11.4	75	HOAD activity	=
Ou [26]	2004	Rat	edl	5,500 m	11.0	90	Palmitate uptake	**↓**
							Palmitate oxidation	**↑**

↑ Change in biomarker is indicative of an *increase* in glycolysis in hypoxia.

= No change in biomarker in hypoxia.

↓ Change in biomarker is indicative of a *decrease* in glycolysis in hypoxia.

*Abbreviations:* *edl extensor digitorum longus*, *mix* mixed skeletal*, pla plantaris*, *rq* red *quadriceps*, *sol soleus*, *vl vastus lateralis*, *wq* white *quadriceps.*

#### TCA cycle

For biomarkers of TCA cycle function, 29 hypoxic settings were identified across 20 papers, the results of which are summarised in Table 4. A decrease in biomarkers of TCA cycle activity was measured in 3/10 settings in humans [16–18] and 8/19 settings in rodents [14, 23, 27, 28, 34, 37, 38], whilst none reported an increase in either group. Moreover, the loss of TCA cycle enzyme activity appears to be dependent on the degree of hypoxic exposure, with 1/14 (7%), 7/15 (47%) and 3/3 (100%) observations at high, very high and extreme degrees of hypoxia, respectively, showing such a loss. This appears to be unrelated to the particular enzyme assayed with activity of aconitase (1 decreased, 2 unchanged), citrate synthase (5 decreased, 13 unchanged), malate dehydrogenase (2 decreased, 4 unchanged) and succinate dehydrogenase (2 decreased, 3 unchanged) either falling or not changing following hypoxic exposure.

**Table 4:** **The effects of environmental hypoxia on biomarkers of TCA cycle function in skeletal muscle**

***First author***	***Year***	***Organism***	***Muscle model***	***Hypoxia model***	***p(O***_***2***_ ***)***_***min***_ ***(kPa)***	***Duration (d)***	***Marker***	***Change***
Morash [31]	2013	Mouse	mix	13% O_2_	13.1	1	Citrate synthase activity	**=**
							Aconitase activity	**=**
Green [15]	1992	Human	vl	4,300 m	12.8	1	Succinate dehydrogenase activity	**=**
Magalhaes [38]	2005	Mouse	mix	8,500 m	7.4	2	Aconitase activity	**↓**
Pastoris [29]	1995	Rat	gnm	5,860 m	10.1	3	Citrate synthase activity	**↓**
							Malate dehydrogenase activity	**=**
Pastoris [29]	1995	Rat	sol	5,860 m	10.1	3	Citrate synthase activity	**=**
							Malate dehydrogenase activity	**=**
Morash [31]	2013	Mouse	mix	13% O_2_	13.1	7	Citrate synthase activity	**=**
							Aconitase activity	**=**
Dutta [28]	2009	Rat	mix	349 mmHg	10.3	7	Citrate synthase activity	**↓**
							Malate dehydrogenase activity	**↓**
							Succinate dehydrogenase activity	**↓**
Vigano [16]	2008	Human	vl	4,559 m	12.4	8	Aconitase levels	**↓**
							α-ketoglutarate dehydrogenase levels	**↓**
							Malate dehydrogenase levels	**↓**
Chaillou [14]	2013	Rat	pla	5,500 m	11.0	9	Citrate synthase activity	**↓**
De Palma [27]	2007	Rat	gnm	10% O_2_	10.1	14	Aconitase levels	**↓**
							Malate dehydrogenase levels	**↓**
							Pyruvate dehydrogenase levels	**↓**
							Succinyl coenzyme A synthetase levels	**↓**
Young [22]	1984	Human	vl	4,300 m	12.8	18	Malate dehydrogenase activity	**=**
Levett [18]	2012	Human	vl	5,300 m	11.3	19	Citrate synthase levels	**=**
							Citrate synthase expression	**=**
Green [15]	1992	Human	vl	4,300 m	12.8	21	Succinate dehydrogenase activity	**=**
Green [19]	2000	Human	vl	6,194 m	10.1	21	Citrate synthase activity	**=**
Daneshrad [24]	2000	Rat	sol	10% O_2_	10.1	21	Citrate synthase activity	**=**
Takahashi [34]	1993	Rat	pla	10% O_2_	10.1	28	Malate dehydrogenase activity	**↓**
Takahashi [34]	1993	Rat	sol	10% O_2_	10.1	28	Malate dehydrogenase activity	**=**
Beaudry [39]	2010	Mouse	gnm	480 mmHg	13.4	28	Citrate synthase activity	**=**
Wuest [40]	2009	Rat	pla	410 mmHg	11.5	28	Succinate dehydrogenase activity	**=**
Jacobs [36]	2013b	Human	vl	3,454 m	14.2	28	Citrate synthase activity	**=**
Galbes [32]	2008	Rat	q	4,000 m	13.3	35	Citrate synthase activity	**=**
Green [17]	1989	Human	vl	8,848 m	7.1	40	Citrate synthase activity	**↓**
							Succinate dehydrogenase activity	**↓**
Chaillou [14]	2013	Rat	pla	5,500 m	11.0	63	Citrate synthase activity	**↓**
Abdelmalki [23]	1996	Rat	sol	13% O_2_	13.1	64	Citrate synthase activity	**=**
Abdelmalki [23]	1996	Rat	pla	13% O_2_	13.1	64	Citrate synthase activity	**↓**
Abdelmalki [23]	1996	Rat	rq	13% O_2_	13.1	64	Citrate synthase activity	**=**
Abdelmalki [23]	1996	Rat	wq	13% O_2_	13.1	64	Citrate synthase activity	**=**
Levett [18]	2012	Human	vl	8,848 m	7.1	66	Citrate synthase levels	**↓**
Mizuno [33]	2008	Human	vl	5,250 m	11.4	75	Citrate synthase activity	**=**

↑ Change in biomarker is indicative of an *increase* in TCA cycle function in hypoxia.

= No change in biomarker in hypoxia.

↓ Change in biomarker is indicative of a *decrease* in TCA cycle function in hypoxia.

*Abbreviations:* *edl extensor digitorum longus*, *gnm gastrocnemius*, *mix* mixed skeletal, *pla plantaris*, *q quadriceps, rq* red *quadriceps, sol soleus, vl vastus lateralis, wq* white *quadriceps*.

#### Oxidative phosphorylation

For biomarkers of oxidative phosphorylation, 19 hypoxic settings were identified across 14 papers, the results of which are summarised in Table 5. Markers of oxidative phosphorylation decreased in 3/4 human settings [16, 18, 36] and 8/15 rodent settings [14, 25, 27, 29, 38, 41], with an increase in 1 of the 15 rodent settings [42]. Complexes I [18, 27], III [16], IV [18], V [16, 18, 27] and the electron-transferring flavoprotein [16] were each shown to be diminished after exposure in various studies. Respirometry performed at high altitude revealed a decrease in oxidative capacity in the presence of both complexes I and II substrates [36].

**Table 5:** **The effects of environmental hypoxia on biomarkers of oxidative phosphorylation in skeletal muscle**

***First author***	***Year***	***Organism***	***Muscle model***	***Hypoxia model***	***p(O***_***2***_ ***)***_***min***_ ***(kPa)***	***Duration (d)***	***Marker***	***Change***
Morash [31]	2013	Mouse	mix	13% O_2_	13.1	1	Complex I OXPHOS	**=**
							Complex II OXPHOS	**=**
							Complex IV OXPHOS	**=**
Magalhaes [38]	2005	Mouse	mix	8,500 m	7.4	2	Complex II OXPHOS	**↓**
Pastoris [29]	1995	Rat	sol	5,860 m	10.1	3	Complex III activity	**=**
							Complex IV activity	**=**
Pastoris [29]	1995	Rat	gnm	5,860 m	10.1	3	Complex III activity	**=**
							Complex IV activity	**↓**
Morash [31]	2013	Mouse	mix	13% O_2_	13.1	7	Complex I OXPHOS	**=**
							Complex II OXPHOS	**=**
							Complex IV OXPHOS	**=**
Vigano [16]	2008	Human	vl	4,559 m	12.4	8	Complex III levels	**↓**
							Complex V levels	**↓**
							ETF levels	**↓**
Chaillou [14]	2013	Rat	pla	5,500 m	11.0	9	Complex IV activity	**↓**
Jacobs	2013a	Human	vl		12.4	10	Complex I OXPHOS	**=**
							Complex II OXPHOS	**=**
							Complex I+II OXPHOS	**=**
De Palma [27]	2007	Rat	gnm	10% O_2_	10.1	14	Complex V levels	**↓**
Daneshrad [42]	2001	Rat	sol	10% O_2_	10.1	21	OXPHOS	**↑**
Beaudry [39]	2010	Mouse	gnm	480 mmHg	13.4	28	Complex IV activity	**=**
Gamboa [41]	2010	Mouse	gnm	10% O_2_	10.1	28	Complex II levels	**↓**
							Complex IV levels	**↓**
							Complex V levels	**↓**
Gamboa [43]	2012	Mouse	mix	10% O_2_	10.1	28	Complex IV levels	**↓**
							Complex V activity	**↑**
							Complex I OXPHOS	**↓**
Jacobs [36]	2013b	Human	vl	3,454 m	14.2	28	Complex I OXPHOS	**↓**
							Complex II OXPHOS	**↓**
							Complex I+II OXPHOS	**↓**
							Complex IV activity	**=**
McClelland [25]	2002	Rat	sol	4,300 m	12.8	56	Complex IV activity	**↓**
McClelland [25]	2002	Rat	pla	4,300 m	12.8	56	Complex IV activity	**↓**
McClelland [25]	2002	Rat	gnm	4,300 m	12.8	56	Complex IV activity	**=**
Chaillou [14]	2013	Rat	pla	5,500 m	11.0	63	Complex IV activity	**↓**
Levett [18]	2012	Human	vl	8,848 m	7.1	66	Complex I expression	**=**
							Complex I levels	**↓**
							Complex II levels	**=**
							Complex III levels	**=**
							Complex IV expression	**=**
							Complex IV levels	**↓**
							Complex V expression	**=**
							Complex V levels	**↓**

↑ Change in biomarker is indicative of an *increase* in oxidative phosphorylation in hypoxia.

= No change in biomarker in hypoxia.

↓ Change in biomarker is indicative of a *decrease* in oxidative phosphorylation in hypoxia.

*Abbreviations:* *gnm gastrocnemius*, *mix* mixed skeletal, *pla plantaris*, *sol soleus*, *vl vastus lateralis.*

#### Mitochondrial density

For biomarkers of mitochondrial density, 34 hypoxic settings were identified across 23 papers, the results of which are summarised in Table 6. Considering only direct observations of mitochondrial density in human *m. vastus lateralis*, 19 d at 5.300 m [18] and 40 d progressive decompression to the equivalent of 8,000 m [44] proved insufficient to induce detectable changes, whilst 56 d at 5,000 m [45] and 66 d spend above 6,600 m [18] resulted in a decrease in mitochondrial density. Considering all biomarkers of mitochondrial density, 4/13 (31%) measures at high, 6/14 (43%) measures at very high and 4/7 (57%) measures in extreme hypoxia, resulted in a significant decrease in biomarkers compared with baseline.

**Table 6:** **The effects of environmental hypoxia on biomarkers of mitochondrial density in skeletal muscle**

***First author***	***Year***	***Organism***	***Muscle model***	***Hypoxia model***	***p(O***_***2***_ ***)***_***min***_ ***(kPa)***	***Duration (d)***	***Marker***	***Change***
Morash [31]	2013	Mouse	mix	13% O_2_	13.1	1	Citrate synthase activity	**=**
							Complex IV OXPHOS	**=**
Magalhaes [38]	2005	Mouse	mix	8,500 m	7.4	2	Complex II OXPHOS	**↓**
Magalhaes [46]	2007	Mouse	mix	8,500 m	7.4	2	Bax expression	**↑**
							Bcl-2 expression	**↓**
Pastoris [29]	1995	Rat	sol	5,860 m	10.1	3	Complex IV activity	**=**
							Citrate synthase activity	**=**
Pastoris [29]	1995	Rat	gnm	5,860 m	10.1	3	Complex IV activity	**↓**
							Citrate synthase activity	**↓**
Chaillou [14]	2013	Rat	pla	5,500 m	11.0	3	BNIP3 expression	**=**
							PGC-1α expression	**=**
Morash [31]	2013	Mouse	mix	13% O_2_	13.1	7	Citrate synthase activity	**=**
							Complex IV OXPHOS	**=**
Dutta [28]	2009	Rat	mix	349 mmHg	10.3	7	Citrate synthase activity	**↓**
Chaillou [14]	2013	Rat	pla	5,500 m	11.0	9	Complex IV activity	**↓**
							Citrate synthase activity	**↓**
							PGC-1α expression	**↑**
							BNIP3 expression	**=**
Jacobs [35]	2013a	Human	vl	4,559 m	12.4	10	Complex I OXPHOS capacity	**=**
							Complex II OXPHOS capacity	**=**
							Complex I+II OXPHOS capacity	**=**
Levett [18]	2012	Human	vl	5,300 m	11.3	19	**Mitochondrial density**	**=**
							PGC-1α levels	**=**
Green [19]	2000	Human	vl	6,194 m	10.1	21	Citrate synthase activity	**=**
Daneshrad [24]	2000	Rat	sol	10% O_2_	10.1	21	Citrate synthase activity	**=**
Daneshrad [42]	2001	Rat	sol	10% O_2_	10.1	21	OXPHOS	**↑**
Beaudry [39]	2010	Mouse	gnm	480 mmHg	13.4	28	Complex IV activity	**=**
							Citrate synthase activity	**=**
Gamboa [41]	2010	Mouse	gnm	10% O_2_	10.1	28	**Mitochondrial density**	**=**
							BNIP3 expression	**=**
							Complex IV levels	**↓**
							PGC-1α levels	**=**
Gamboa [43]	2012	Mouse	mix	10% O_2_	10.1	28	Complex I OXPHOS	**↓**
Jacobs [36]	2013b	Human	vl	3,454 m	14.2	28	Complex I OXPHOS	**↓**
							Complex II OXPHOS	**↓**
							Complex I+II OXPHOS	**↓**
							Complex IV activity	**=**
							Citrate synthase activity	**=**
Galbes [32]	2008	Rat	q	4,000 m	13.3	35	Citrate synthase activity	**=**
Green [17]	1989	Human	vl	8,848 m	7.1	40	Citrate synthase activity	**↓**
MacDougall [44]	1991	Human	vl	8,848 m	7.1	40	**Mitochondrial density**	**=**
van Ekeren [47]	1992	Rat	edl	8% O_2_	8.1	45	**Mitochondrial density**	**↑**
van Ekeren [47]	1992	Rat	sol	8% O_2_	8.1	45	**Mitochondrial density**	**↓**
Hoppeler [45]	1990	Human	vl	5,000 m	11.7	56	**Mitochondrial density**	**↓**
McClelland [25]	2002	Rat	sol	4,300 m	12.8	56	Complex IV activity	**↓**
McClelland [25]	2002	Rat	gnm	4,300 m	12.8	56	Complex IV activity	**=**
McClelland [25]	2002	Rat	pla	4,300 m	12.8	56	Complex IV activity	**↓**
Chaillou [14]	2013	Rat	pla	5,500 m	11.0	63	Complex IV activity	**↓**
							Citrate synthase activity	**↓**
Abdelmalki [23]	1996	Rat	sol	13% O_2_	13.1	64	Citrate synthase activity	**=**
Abdelmalki [23]	1996	Rat	pla	13% O_2_	13.1	64	Citrate synthase activity	**↓**
Abdelmalki [23]	1996	Rat	rq	13% O_2_	13.1	64	Citrate synthase activity	**=**
Abdelmalki [23]	1996	Rat	wq	13% O_2_	13.1	64	Citrate synthase activity	**=**
Levett [18]	2012	Human	vl	8,848 m	7.1	66	**Mitochondrial density**	**↓**
							PGC-1α levels	**↓**
							PGC-1α expression	**=**
Mizuno [33]	2008	Human	vl	5,250 m	11.4	75	Citrate synthase activity	**=**

↑ Change in biomarker is indicative of an *increase* in mitochondrial density in hypoxia.

= No change in biomarker in hypoxia.

↓ Change in biomarker is indicative of a *decrease* in mitochondrial density in hypoxia.

*Abbreviations:* *gnm gastrocnemius*, *mix* mixed skeletal, *pla plantaris*, *q quadriceps*, *rq* red *quadriceps, sol soleus, vl vastus lateralis, wq* white *quadriceps*.

#### Summary of results

**Figure 3:**
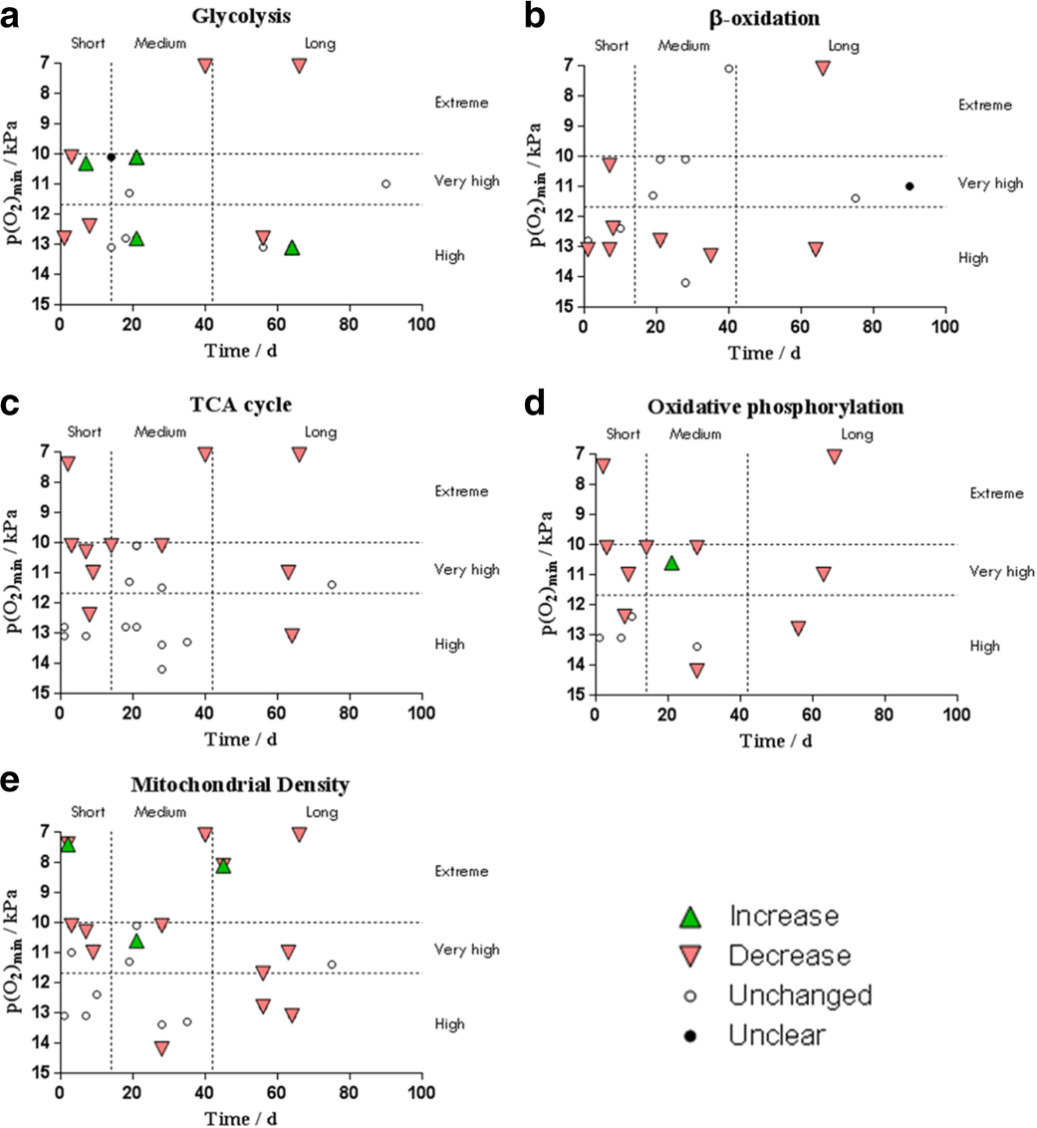
**The effects of environmental hypoxia, in studies of rodent and human skeletal muscle, on (a) glycolysis, (b) β-oxidation, (c) TCA cycle, (d) oxidative phosphorylation and (e) mitochondrial density with varying duration and estimated environmental p(O**_**2**_ **) of the hypoxic setting.** *Increase* indicates settings where at least one biomarker of the process was significantly increased by hypoxia and none decreased; *decrease* indicates settings where at least one biomarker of the process was significantly decreased by hypoxia and none increased; *unchanged* indicates settings where no biomarker was significantly altered by hypoxia; and *unclear* indicates settings where at least one biomarker was increased and another decreased by hypoxia.

**Figure 4:**
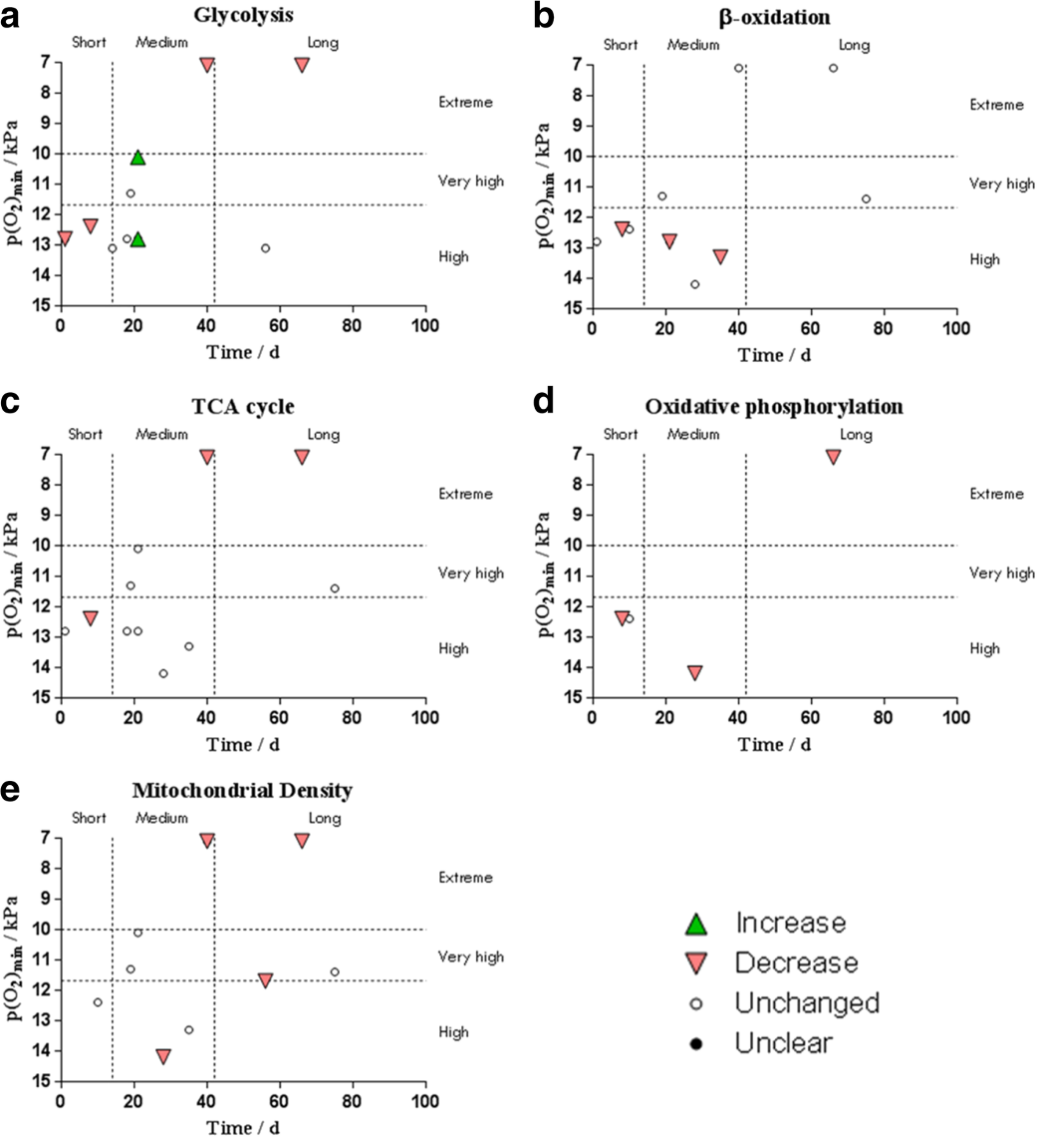
**The effects of environmental hypoxia, in human** ***m. vastus lateralis only*** **, on (a) glycolysis, (b) β-oxidation, (c) TCA cycle, (d) oxidative phosphorylation and (e) mitochondrial density with varying duration and estimated environmental p(O**_**2**_ **) of the hypoxic setting.** *Increase* indicates settings where at least one biomarker of the process was significantly increased by hypoxia and none decreased; *decrease* indicates settings where at least one biomarker of the process was significantly decreased by hypoxia and none increased; *unchanged* indicates settings where no biomarker was significantly altered by hypoxia; and *unclear* indicates settings where at least one biomarker was increased and another decreased by hypoxia.

The effect of each hypoxic setting on glycolysis, β-oxidation, TCA cycle, oxidative phosphorylation and mitochondrial density is represented graphically in Figure 3, for all organisms and in Figure 4 for human *m. vastus lateralis* only.

### Discussion

In this review, we set out to understand the remodelling of metabolic processes in the mammalian skeletal muscle *in vivo* in response to environmental hypoxia, accounting for variations in degree and duration of hypoxic exposure. To do so, we reviewed the literature considering a broad range of biomarkers pertinent to mitochondrial energy metabolism and glycolysis and collated the results to gauge whether a consensus exists within the literature. Whilst both human and rodent studies were included, we initially considered all findings together for completion, followed by data from human *m. vastus lateralis* in isolation for clarity.

Environmental hypoxia induces a loss of mitochondrial density in human *m. vastus lateralis* after long-term [18, 48] but not short-term [35] exposure. Although studies involving adapted populations were excluded from our analysis, it is interesting to note that the skeletal muscle of highland Tibetans is less rich in mitochondria than that of lowlanders [49], as this supports the idea that this is an adaptive trait. Attenuation of oxidative processes, such as β-oxidation [16, 18, 20, 23, 28, 31, 32], the TCA cycle [14, 16, 17, 23, 27–29, 34, 38] and oxidative phosphorylation [14, 16, 18, 25, 27, 29, 36, 38, 41], also seems to be induced by environmental hypoxia. The effect of hypoxia on glycolytic capacity is less clear, with some studies showing increased [19, 20] and others decreased [15–18] levels of biomarkers.

The hypoxia-induced downregulation of β-oxidation, TCA cycle function and oxidative phosphorylation may be secondary to a loss of mitochondrial density, as in short-term (≤14 d) hypoxic settings, all were diminished in at least some studies of human *m. vastus lateralis*, whilst mitochondrial density remained unchanged (Table 7). Some medium-term (≤42 d) and most long-term (>42 d) settings resulted in a significant loss of skeletal muscle mitochondrial density. This therefore suggests that hypoxia-induced remodelling of mitochondrial pathways precedes a loss of mitochondrial density*.* This notion receives support from Jacobs and colleagues, who measured a loss of oxidative capacity, which persisted when respiration was corrected to citrate synthase activity [36], an established marker of mitochondrial density in human muscle [13]. A possible mechanism underpinning this might be that the mismatch in oxygen supply and demand results in ROS production at complexes I and III. This ROS production within the mitochondrion may result in damage to intra-mitochondrial machinery and thus result in loss of function. Alternatively, ROS are known to stabilise HIF, which in the long term may induce changes in mitochondrial density (through BNIP3 and PGC1α) [6, 48] and muscle mass, but may also remodel metabolic pathways in the short term. Indeed, complex I and aconitase, an enzyme of the TCA cycle, are known to be particularly susceptible to HIF-mediated loss of function via miR-210 upregulation [50, 51].

**Table 7:** **Time course of hypoxic response**

Duration	Glycolysis			β-oxidation			TCA cycle function			Oxidative phosphorylation			Mitochondrial density		
	↑	=	↓	↑	=	↓	↑	=	↓	↑	=	↓	↑	=	↓
Short	0%	50%	50%	0%	75%	25%	0%	50%	50%	0%	50%	50%	0%	100%	0%
Medium	33%	50%	17%	0%	67%	33%	0%	86%	14%	0%	0%	100%	0%	60%	40%
Long	0%	50%	50%	0%	50%	50%	0%	50%	50%	0%	0%	100%	0%	33%	67%

The percentage of hypoxic settings in which biomarkers report a significant decrease (**↓**), a significant increase (**↑**) or unchanged/unclear results (**=**) in human *m. vastus lateralis*, following short- (0–14 d), medium- (15–42 d) or long- (43–90 d) term exposure to an environmental p(O_2_) <15 kPa.

It has been hypothesised that environmental hypoxia could alter the balance of substrate utilisation, with an enhanced use of carbohydrates and a correspondingly diminished use fatty acids [11]. Indeed in the hypoxic rat heart, a downregulation of fatty acid oxidation has been reported [52, 53]. Such a substrate switch would be expected to be beneficial, as the oxidation of fatty acids requires more O_2_ per ATP synthesised than the complete oxidation of carbohydrates [54]; thus, an increased reliance on carbohydrates may improve oxygen efficiency. If such a hypoxia-induced switch did occur, it might be expected that biomarkers for β-oxidation would be attenuated more frequently than biomarkers for oxidative phosphorylation. However, this does not appear to be the case, as 8/22 (36%) hypoxic settings induced a significant decrease in a biomarker of β-oxidation whilst 11/19 (58%) altered oxidative phosphorylation. Of those settings in which biomarkers of both β-oxidation and oxidative phosphorylation were measured, 1/4 showed a decrease in oxidative phosphorylation with no change in β-oxidation [36], 2/4 showed a decrease in both [16, 18] and 1/4 reported no change in either [35]. Work from our laboratory in rat soleus found that oxygen consumption in the presence of an acyl-carnitine was lower following hypoxic exposure, whilst respiration when complexes I and II were activated directly was unaltered [31], which is indicative of a substrate switch. In humans, however, the opposite was found to be true, as acyl-carnitine-driven oxygen consumption was unchanged by hypoxia, whilst complex I + II-driven respiration was diminished [36]. Roberts *et al.* showed that 21 d at 4,300 m increased glucose uptake [20] and decreased fatty acid oxidation [30] in human *m. vastus lateralis*. It is unclear, however, whether this increase in glucose uptake supported increased lactate production through lactate dehydrogenase (LDH) or pyruvate oxidation via pyruvate dehydrogenase (PDH) and the TCA cycle. Research into PDH activity following hypoxic exposure is limited, though LDH activity has been reported to rise following hypoxic exposure in humans [19] and rats [28]. A direct comparison of activities of LDH and PDH following hypoxia would be revealing.

Whilst oxidative processes are selectively downregulated in the skeletal muscle following exposure to environmental hypoxia, in contrast to studies in cultured cells, glycolytic markers appear to remain largely unchanged. It is noteworthy, however, that there has been a distinct lack of direct measurements of glycolytic flux *in vivo* or *ex vivo* following hypoxic exposure. These would be revealing, as glycolytic flux can increase in skeletal muscle by up to 1,000-fold upon the onset of high-intensity exercise [55]. Resting glycolytic flux is thus significantly below capacity, and as such measures of capacity, by protein expression or enzyme activity, would not accurately reflect flux *in vivo* at normal levels of exertion. Even so, our analysis of biomarkers of glycolytic capacity suggests that the relative contribution of glycolytic *versus* oxidative ATP production is increased by a hypoxic stimulus and this might be exaggerated upon exertion. An increased dependence on glycolysis would improve oxygen economy but would limit the scope for ATP production in the respiring muscle and result in inefficient use of fuel reserves. The ‘lactate paradox’ originally described by West [56] states that short-term environmental hypoxia does not alter concentrations of blood lactate ([La_b_]) during any given submaximal exercise workload, yet work capacity decreases markedly in hypoxic environments; hence, [La_b_] is lower at maximal workloads. The literature might support this assertion, as glycolytic flux is on the whole unaffected by hypoxic exposure. Today, the lactate paradox is more commonly defined as the phenomenon in which an acute sojourn at altitude induces an increase in blood-lactate accumulation during exercise in the short term, yet this decreases after chronic exposure [21, 57, 58]. However, whilst this may reflect some aspect of metabolic remodelling following hypoxic acclimation, current explanations for this phenomenon remain controversial and probably involve factors beyond the mere capacity for substrate utilisation [59, 60].

The primary strength of our approach is that we provide a thorough and, as far as possible, objective analysis of the literature to date. By collating the available data from a range of animal models and different muscles, it is easy to identify clear, repeatable trends in the effects of environmental hypoxia on aspects of skeletal muscle energy metabolism. Moreover, the exclusion of datasets with confounding factors (e.g. explicit exercise training or pharmacological therapy) maximises the likelihood that these trends are a consequence of environmental hypoxia alone, with the caveat that a sojourn to altitude in itself inevitably introduces confounding variables other than hypoxia, e.g. cold, altered nutrition and possibly infection or gastrointestinal upset. Organising observations of biomarkers into hypoxic ‘settings’ allows for the fact that these observations are unlikely to be independent and sub-categorising these settings by duration and degree of hypoxic exposure and human *versus* rodent studies gives insight into the process of acclimation to hypoxic environments.

There are, however, a number of limitations to the methods used in this review. First, a wide range of animal and muscle models were accepted for analysis in this review, which, whilst a strength in itself, would have led to the inclusion of a number of different control groups across different studies, introducing baseline variation. Second, the time-dependence of rodent and human responses would likely be different, though we have considered data from human *m. vastus lateralis* separately where possible. Third, metabolic studies of muscles are beset by confounding factors relating to prior training status, species, fibre types and possibly even the specific skeletal muscle studied [61, 62]. Fourth, whilst hypoxic settings taken from the same study are treated as independent in this review, the same equipment, experimenters and techniques were most likely used in each setting and thus a directional change in a biomarker might be more likely to be observed in two settings from the same paper than in two settings from different papers. Indeed, five rodent studies looked at different muscles presumably within the same animals in most cases, generating multiple settings (by our definition) which were clearly not independent. An alternative approach might have arbitrarily excluded one or more sets of data or attempted to combine findings or find consensus across different muscles; however, these approaches would each have been problematic in terms of presenting a complete set of findings or introducing bias.

## Conclusions

The literature suggests that skeletal muscle oxidative metabolism is lowered by exposure to environmental hypoxia, which may precede a loss in muscle mitochondrial density. Meanwhile, the total capacity for skeletal muscle glycolysis is not consistently altered by environmental hypoxia. Taken together, the literature is not clear on whether a hypoxia-induced substrate switch from fatty acid oxidation to glucose oxidation occurs within the mitochondria of skeletal muscle as it does in the hypoxic rat heart, for instance. Environmental hypoxia does however induce a selective attenuation of whole muscle fatty acid oxidation, whilst glucose uptake is maintained or increased, perhaps to support glycolytic flux in the face of a downregulation of oxidative metabolism, optimising the pathways of ATP synthesis for the hypoxic environment.

## Authors’ information

AJM and JAH are members of the Caudwell Xtreme Everest Oxygen Research Consortium.

## Electronic supplementary material

Additional file 1: Table S1: A list of all articles reviewed, their inclusion status and reasons for exclusion, where applicable. (DOCX 159 KB)

## Abbreviations

Edl: *Extensor digitorum longus*
gnm: *Gastrocnemius*
mix: *Mixed skeletal*
pla: *Plantaris*
rq: *Red quadriceps*
sol: *Soleus*
vl: *Vastus lateralis*
wq: *White quadriceps*
ADP: Adenosine diphosphate
ATP: Adenosine triphosphate
Bax: Bcl-2-associated X protein
Bcl-2: B-cell lymphoma 2
BNIP3: BCL2/adenovirus E1B protein-interacting protein 3
CACT: Carnitine acylcarnitine translocase
CPT: Carnitine palmitoyl transferase
ECAH: Enoyl CoA hydratase
ECAI: Enoyl CoA isomerase
ETF: Electron-transferring flavoprotein
HIF: Hypoxia-inducible factor
HOAD: L-3-hydroxyacyl CoA dehydrogenase
LDH: Lactate dehydrogenase
OXPHOS: Oxidative phosphorylation
PDH: Pyruvate dehydrogenase
PGC1α: Peroxisome proliferator-activated receptor gamma coactivator 1-alpha
PPARα: Peroxisome proliferator-activated receptor alpha
ROS: Reactive oxygen species
TCA: Tricarboxylic acid.
