# Supplementary material for: Skeletal muscle energy metabolism in environmental hypoxia: climbing towards consensus
Source: Extrem Physiol Med. 2014 Nov 28;3:19. doi: 10.1186/2046-7648-3-19 (PMC4253994; doi:10.1186/2046-7648-3-19)
Supplement: Supplementary file 1 — Additional file 1: Table S1: A list of all articles reviewed, their inclusion status and reasons for exclusion, where applicable. (DOCX 159 KB) [file 13728_2014_67_MOESM1_ESM.docx]

**Skeletal muscle energy metabolism in environmental hypoxia: climbing towards consensus**

**Online Supplement**

James A. Horscroft and Andrew J. Murray

*Department of Physiology, Development and Neuroscience, University of Cambridge, Downing Street, Cambridge, United Kingdom*

**Corresponding author:**

James A. Horscroft
Department of Physiology, Development and Neuroscience
University of Cambridge
Downing Street
CB2 3EG
United Kingdom
jah212@cam.ac.uk

**Table S1. A list of all articles reviewed, their inclusion status and reasons for exclusion, where applicable.**

| Ref. | First author | Year | Included/Excluded | Reason for exclusion |
| --- | --- | --- | --- | --- |
| [1] | Gold | 1973 | Excluded | Skeletal muscle not assessed. |
| [2] | Berlet | 1975 | Excluded | Skeletal muscle not assessed. |
| [3] | Gimenez | 1975 | Excluded | Non-mammalian model. |
| [4] | Andersson | 1979 | Excluded | No environmental hypoxia. |
| [5] | Shmerling | 1982 | Excluded | Confounded by exercise. |
| [6] | Sahlin | 1983 | Excluded | No environmental hypoxia. |
| [7] | **Young** | **1984** | **Included** | **-** |
| [8] | Millis | 1984 | Excluded | No relevant biomarker assessed. |
| [9] | Robin | 1984 | Excluded | Non-mammalian model. |
| [10] | Pastoris | 1985 | Excluded | Non-continuous hypoxia. |
| [11] | Millis | 1985 | Excluded | No environmental hypoxia. |
| [12] | **Green** | **1989** | **Included** | **-** |
| [13] | Howald | 1990 | Excluded | Confounded by exercise. |
| [14] | **Hoppeler** | **1990** | **Included** | **-** |
| [15] | Martinelli | 1990 | Excluded | No relevant biomarker assessed. |
| [16] | Katz | 1990 | Excluded | No environmental hypoxia. |
| [17] | Cartee | 1991 | Excluded | Confounded by CO_2_. |
| [18] | Bigard | 1991 | Excluded | Confounded by exercise. |
| [19] | **MacDougall** | **1991** | **Included** | **-** |
| [20] | Kayser | 1991 | Excluded | No relevant biomarker assessed. |
| [21] | **Green** | **1992** | **Included** | **-** |
| [22] | **van Ekeren** | **1992** | **Included** | **-** |
| [23] | **Takahashi** | **1993** | **Included** | **-** |
| [24] | Desplanches | 1993 | Excluded | Confounded by exercise. |
| [25] | Semenza | 1994 | Excluded | Non-mammalian model. |
| [26] | Azevedo | 1995 | Excluded | No relevant biomarker assessed. |
| [27] | **Pastoris** | **1995** | **Included** | **-** |
| [28] | **Roberts** | **1996a** | **Included** | **-** |
| [29] | **Roberts** | **1996b** | **Included** | **-** |
| [30] | Kayser | 1996 | Excluded | Confounded by adapted human population. |
| [31] | Punkt | 1996 | Excluded | <1 d hypoxic exposure. |
| [32] | **Abdelmalki** | **1996** | **Included** | **-** |
| [33] | Pastoris | 1998 | Excluded | No relevant biomarker assessed. |
| [34] | van der Laan | 1998 | Excluded | No environmental hypoxia. |
| [35] | Garry | 1998 | Excluded | Confounded by genetic mouse model. |
| [36] | Kanatous | 1999 | Excluded | Non-mammalian model. |
| [37] | Richardson | 1999 | Excluded | Confounded by exercise. |
| [38] | McClelland | 1999 | Excluded | Confounded by exercise. |
| [39] | Lundby | 2000 | Excluded | Confounded by exercise. |
| [40] | St-Pierre | 2000a | Excluded | Non-mammalian model. |
| [41] | St-Pierre | 2000b | Excluded | Non-mammalian model. |
| [42] | Zhou | 2000 | Excluded | No relevant biomarker assessed. |
| [43] | **Daneshrad** | **2000** | **Included** | **-** |
| [44] | St-Pierre | 2000c | Excluded | Non-mammalian model. |
| [45] | Parolin | 2000a | Excluded | Confounded by exercise. |
| [46] | Parolin | 2000b | Excluded | Confounded by exercise. |
| [47] | **Green** | **2000** | **Included** | **-** |
| [48] | Jaakkola | 2001 | Excluded | Non-mammalian model. |
| [49] | **Daneshrad** | **2001** | **Included** | **-** |
| [50] | Lin | 2002 | Excluded | No environmental hypoxia. |
| [51] | **McClelland** | **2002** | **Included** | **-** |
| [52] | Kubasiak | 2002 | Excluded | Non-mammalian model. |
| [53] | Kanatous | 2002 | Excluded | Non-mammalian model. |
| [54] | Howald | 2003 | Excluded | Returned to normoxia before sampling. |
| [55] | Pyner | 2003 | Excluded | No relevant biomarker assessed. |
| [56] | Juel | 2003 | Excluded | No relevant biomarker assessed. |
| [57] | Stavinoha | 2004 | Excluded | No relevant biomarker assessed. |
| [58] | **Ou** | **2004** | **Included** | **-** |
| [59] | Lundby | 2004 | Excluded | No relevant biomarker assessed. |
| [60] | Mason | 2004 | Excluded | No environmental hypoxia. |
| [61] | Gelfi | 2004 | Excluded | Confounded by adapted human population. |
| [62] | **Magalhaes** | **2005** | **Included** | **-** |
| [63] | Ponsot | 2006 | Excluded | Confounded by exercise. |
| [64] | Navet | 2005 | Excluded | No environmental hypoxia. |
| [65] | Ripamonti | 2006 | Excluded | Non-continuous hypoxia. |
| [66] | Lee | 2006 | Excluded | No environmental hypoxia. |
| [67] | Martinez | 2006 | Excluded | Non-mammalian model. |
| [68] | Fukuda | 2007 | Excluded | Non-mammalian model. |
| [69] | Roels | 2007 | Excluded | Confounded by exercise. |
| [70] | **De Palma** | **2007** | **Included** | **-** |
| [71] | Bakkman | 2007 | Excluded | Confounded by exercise. |
| [72] | **Magalhaes** | **2007** | **Included** | **-** |
| [73] | Haseler | 2007 | Excluded | Confounded by exercise. |
| [74] | **Vigano** | **2008** | **Included** | **-** |
| [75] | **Galbes** | **2008** | **Included** | **-** |
| [76] | Aragones | 2008 | Excluded | No environmental hypoxia. |
| [77] | Zhang | 2008 | Excluded | Non-mammalian model. |
| [78] | Esteva | 2008 | Excluded | No environmental hypoxia. |
| [79] | Lu | 2008 | Excluded | Non-mammalian model. |
| [80] | Dash | 2008 | Excluded | Non-mammalian model. |
| [81] | Bo | 2008 | Excluded | <1 d hypoxic exposure. |
| [82] | **Mizuno** | **2008** | **Included** | **-** |
| [83] | O’Hagan | 2008 | Excluded | No environmental hypoxia. |
| [84] | **van Hall** | **2009** | **Included** | **-** |
| [85] | Chan | 2009 | Excluded | No relevant biomarker assessed. |
| [86] | **Dutta** | **2009** | **Included** | **-** |
| [87] | Scott | 2009 | Excluded | Non-mammalian model. |
| [88] | Straadt | 2009 | Excluded | Non-mammalian model. |
| [89] | **Wuest** | **2009** | **Included** | **-** |
| [90] | **Gamboa** | **2010** | **Included** | **-** |
| [91] | Ponsot | 2010 | Excluded | Confounded by exercise. |
| [92] | Schroeder | 2010 | Excluded | No relevant biomarker assessed. |
| [93] | Schmutz | 2010 | Excluded | Confounded by exercise. |
| [94] | Saxena | 2010 | Excluded | No environmental hypoxia. |
| [95] | **Beaudry** | **2010** | **Included** | **-** |
| [96] | Dasika | 2010 | Excluded | No environmental hypoxia. |
| [97] | Li | 2010 | Excluded | Confounded by exercise. |
| [98] | Pesta | 2011 | Excluded | Confounded by exercise. |
| [99] | Fusco | 2011 | Excluded | No environmental hypoxia. |
| [100] | Heinonen | 2011 | Excluded | Confounded by exercise. |
| [101] | **Gamboa** | **2012** | **Included** | **-** |
| [102] | **Levett** | **2012** | **Included** | **-** |
| [103] | Lin | 2012 | Excluded | Non-mammalian model. |
| [104] | Robach | 2012 | Excluded | Confounded by exercise. |
| [105] | Chen | 2013 | Excluded | Non-mammalian model. |
| [106] | **Jacobs** | **2013a** | **Included** | **-** |
| [107] | **Jacobs** | **2013b** | **Included** | **-** |
| [108] | **Chaillou** | **2013** | **Included** | **-** |
| [109] | Wang | 2013 | Excluded | Non-mammalian model. |
| [110] | Gomes | 2013 | Excluded | No environmental hypoxia. |
| [111] | **Morash** | **2013** | **Included** | **-** |
| [112] | Dromparis | 2014 | Excluded | No environmental hypoxia. |
| [113] | Thom | 2014 | Excluded | No relevant biomarker assessed. |

**Number of papers included: 33**

**Number of papers excluded: 80**

1. <1 d hypoxic exposure. 2
2. Confounded by adapated human population. 2
3. Confounded by CO_2._ 1
4. Confounded by exercise. 19
5. Confounded by genetic mouse model. 1
6. No environmental hypoxia. 17
7. No relevant biomarker assessed. 13
8. Non-continuous hypoxia. 3
9. Non-mammalian model. 20
10. Skeletal muscle not assessed. 2

1. Gold AJ, Johnson TF, Costello LC: **Effects of altitude stress on mitochondrial function.** *Am J Physiol* 1973, **224:**946-949.

2. Berlet HH: **Uptake and phosphorylation of (14C) creatine by mouse cardiac muscle in vivo.** *Recent Adv Stud Cardiac Struct Metab* 1975, **7:**183-192.

3. Gimenez M, Sanderson RJ, Reiss OK, Banchero N: **Effects of altitude on myoglobin and mitochondrial protein in canine skeletal muscle.** *Respiration* 1977, **34:**171-176.

4. Andersson J, Eklof B, Neglen P, Thomson D: **Metabolic changes in blood and skeletal muscle in reconstructive aortic surgery.** *Ann Surg* 1979, **189:**283-289.

5. Shmerling MD, Filiushina EE, Buzueva, II: **[Electron microscope study of skeletal muscle fibers during physical exercise in high-altitude hypoxia].** *Biull Eksp Biol Med* 1982, **94:**119-122.

6. Sahlin K: **NADH and NADPH in human skeletal muscle at rest and during ischaemia.** *Clin Physiol* 1983, **3:**477-485.

7. Young AJ, Evans WJ, Fisher EC, Sharp RL, Costill DL, Maher JT: **Skeletal muscle metabolism of sea-level natives following short-term high-altitude residence.** *Eur J Appl Physiol Occup Physiol* 1984, **52:**463-466.

8. Millis RM, Stephens TA, Harris G, Anonye C, Reynolds M: **Relationship between intracellular oxygenation and neuromuscular conduction during hypoxic hypoxia.** *Life Sci* 1984, **35:**2443-2451.

9. Robin ED, Murphy BJ, Theodore J: **Coordinate regulation of glycolysis by hypoxia in mammalian cells.** *J Cell Physiol* 1984, **118:**287-290.

10. Pastoris O, Dossena M, Foppa P, Arnaboldi R, Gorini A, Villa RF, Benzi G: **Modifications by chronic intermittent hypoxia and drug treatment on skeletal muscle metabolism.** *Neurochem Res* 1995, **20:**143-150.

11. Millis RM, Stephens TA, Harris G, Anonye C, Reynolds M: **Histochemical assessment of cytochrome oxidase activity for monitoring ischemic muscle injury.** *Exp Neurol* 1985, **88:**265-276.

12. Green HJ, Sutton JR, Cymerman A, Young PM, Houston CS: **Operation Everest II: adaptations in human skeletal muscle.** *J Appl Physiol (1985)* 1989, **66:**2454-2461.

13. Howald H, Pette D, Simoneau JA, Uber A, Hoppeler H, Cerretelli P: **Effect of chronic hypoxia on muscle enzyme activities.** *Int J Sports Med* 1990, **11 Suppl 1:**S10-14.

14. Hoppeler H, Kleinert E, Schlegel C, Claassen H, Howald H, Kayar SR, Cerretelli P: **Morphological adaptations of human skeletal muscle to chronic hypoxia.** *Int J Sports Med* 1990, **11 Suppl 1:**S3-9.

15. Martinelli M, Winterhalder R, Cerretelli P, Howald H, Hoppeler H: **Muscle lipofuscin content and satellite cell volume is increased after high altitude exposure in humans.** *Experientia* 1990, **46:**672-676.

16. Katz A, Spencer MK, Sahlin K: **Failure of glutamate dehydrogenase system to predict oxygenation state of human skeletal muscle.** *Am J Physiol* 1990, **259:**C26-28.

17. Cartee GD, Douen AG, Ramlal T, Klip A, Holloszy JO: **Stimulation of glucose transport in skeletal muscle by hypoxia.** *J Appl Physiol (1985)* 1991, **70:**1593-1600.

18. Bigard AX, Brunet A, Guezennec CY, Monod H: **Skeletal muscle changes after endurance training at high altitude.** *J Appl Physiol (1985)* 1991, **71:**2114-2121.

19. MacDougall JD, Green HJ, Sutton JR, Coates G, Cymerman A, Young P, Houston CS: **Operation Everest II: structural adaptations in skeletal muscle in response to extreme simulated altitude.** *Acta Physiol Scand* 1991, **142:**421-427.

20. Kayser B, Hoppeler H, Claassen H, Cerretelli P: **Muscle structure and performance capacity of Himalayan Sherpas.** *J Appl Physiol (1985)* 1991, **70:**1938-1942.

21. Green HJ, Sutton JR, Wolfel EE, Reeves JT, Butterfield GE, Brooks GA: **Altitude acclimatization and energy metabolic adaptations in skeletal muscle during exercise.** *J Appl Physiol (1985)* 1992, **73:**2701-2708.

22. van Ekeren GJ, Sengers RC, Stadhouders AM: **Changes in volume densities and distribution of mitochondria in rat skeletal muscle after chronic hypoxia.** *Int J Exp Pathol* 1992, **73:**51-60.

23. Takahashi H, Kikuchi K, Nakayama H: **Effect of chronic hypoxia on oxidative enzyme activity in rat skeletal muscle.** *Ann Physiol Anthropol* 1993, **12:**363-369.

24. Desplanches D, Hoppeler H, Linossier MT, Denis C, Claassen H, Dormois D, Lacour JR, Geyssant A: **Effects of training in normoxia and normobaric hypoxia on human muscle ultrastructure.** *Pflugers Arch* 1993, **425:**263-267.

25. Semenza GL, Roth PH, Fang HM, Wang GL: **Transcriptional regulation of genes encoding glycolytic enzymes by hypoxia-inducible factor 1.** *J Biol Chem* 1994, **269:**23757-23763.

26. Azevedo JL, Jr., Carey JO, Pories WJ, Morris PG, Dohm GL: **Hypoxia stimulates glucose transport in insulin-resistant human skeletal muscle.** *Diabetes* 1995, **44:**695-698.

27. Pastoris O, Foppa P, Catapano M, Dossena M: **Effects of hypoxia on enzyme activities in skeletal muscle of rats of different ages. An attempt at pharmacological treatment.** *Pharmacol Res* 1995, **32:**375-381.

28. Roberts AC, Reeves JT, Butterfield GE, Mazzeo RS, Sutton JR, Wolfel EE, Brooks GA: **Altitude and beta-blockade augment glucose utilization during submaximal exercise.** *J Appl Physiol (1985)* 1996, **80:**605-615.

29. Roberts AC, Butterfield GE, Cymerman A, Reeves JT, Wolfel EE, Brooks GA: **Acclimatization to 4,300-m altitude decreases reliance on fat as a substrate.** *J Appl Physiol (1985)* 1996, **81:**1762-1771.

30. Kayser B, Hoppeler H, Desplanches D, Marconi C, Broers B, Cerretelli P: **Muscle ultrastructure and biochemistry of lowland Tibetans.** *J Appl Physiol (1985)* 1996, **81:**419-425.

31. Punkt K, Unger A, Welt K, Hilbig H, Schaffranietz L: **Hypoxia-dependent changes of enzyme activities in different fibre types of rat soleus and extensor digitorum longus muscles. A cytophotometrical study.** *Acta Histochem* 1996, **98:**255-269.

32. Abdelmalki A, Fimbel S, Mayet-Sornay MH, Sempore B, Favier R: **Aerobic capacity and skeletal muscle properties of normoxic and hypoxic rats in response to training.** *Pflugers Arch* 1996, **431:**671-679.

33. Pastoris O, Foppa P, Catapano M, Dossena M: **Metabolite concentrations in skeletal muscle of different aged rats submitted to hypoxia and pharmacological treatment with nicergoline.** *Exp Gerontol* 1998, **33:**303-318.

34. van der Laan L, Coremans A, Ince C, Bruining HA: **NADH videofluorimetry to monitor the energy state of skeletal muscle in vivo.** *J Surg Res* 1998, **74:**155-160.

35. Garry DJ, Ordway GA, Lorenz JN, Radford NB, Chin ER, Grange RW, Bassel-Duby R, Williams RS: **Mice without myoglobin.** *Nature* 1998, **395:**905-908.

36. Kanatous SB, DiMichele LV, Cowan DF, Davis RW: **High aerobic capacities in the skeletal muscles of pinnipeds: adaptations to diving hypoxia.** *J Appl Physiol (1985)* 1999, **86:**1247-1256.

37. Richardson RS, Leigh JS, Wagner PD, Noyszewski EA: **Cellular PO2 as a determinant of maximal mitochondrial O(2) consumption in trained human skeletal muscle.** *J Appl Physiol (1985)* 1999, **87:**325-331.

38. McClelland GB, Hochachka PW, Weber JM: **Effect of high-altitude acclimation on NEFA turnover and lipid utilization during exercise in rats.** *Am J Physiol* 1999, **277:**E1095-1102.

39. Lundby C, Saltin B, van Hall G: **The 'lactate paradox', evidence for a transient change in the course of acclimatization to severe hypoxia in lowlanders.** *Acta Physiol Scand* 2000, **170:**265-269.

40. St-Pierre J, Brand MD, Boutilier RG: **The effect of metabolic depression on proton leak rate in mitochondria from hibernating frogs.** *J Exp Biol* 2000, **203:**1469-1476.

41. St-Pierre J, Tattersall GJ, Boutilier RG: **Metabolic depression and enhanced O(2) affinity of mitochondria in hypoxic hypometabolism.** *Am J Physiol Regul Integr Comp Physiol* 2000, **279:**R1205-1214.

42. Zhou M, Lin BZ, Coughlin S, Vallega G, Pilch PF: **UCP-3 expression in skeletal muscle: effects of exercise, hypoxia, and AMP-activated protein kinase.** *Am J Physiol Endocrinol Metab* 2000, **279:**E622-629.

43. Daneshrad Z, Garcia-Riera MP, Verdys M, Rossi A: **Differential responses to chronic hypoxia and dietary restriction of aerobic capacity and enzyme levels in the rat myocardium.** *Mol Cell Biochem* 2000, **210:**159-166.

44. St-Pierre J, Brand MD, Boutilier RG: **Mitochondria as ATP consumers: cellular treason in anoxia.** *Proc Natl Acad Sci U S A* 2000, **97:**8670-8674.

45. Parolin ML, Spriet LL, Hultman E, Hollidge-Horvat MG, Jones NL, Heigenhauser GJ: **Regulation of glycogen phosphorylase and PDH during exercise in human skeletal muscle during hypoxia.** *Am J Physiol Endocrinol Metab* 2000, **278:**E522-534.

46. Parolin ML, Spriet LL, Hultman E, Matsos MP, Hollidge-Horvat MG, Jones NL, Heigenhauser GJ: **Effects of PDH activation by dichloroacetate in human skeletal muscle during exercise in hypoxia.** *Am J Physiol Endocrinol Metab* 2000, **279:**E752-761.

47. Green H, Roy B, Grant S, Otto C, Pipe A, McKenzie D, Johnson M: **Human skeletal muscle exercise metabolism following an expedition to mount denali.** *Am J Physiol Regul Integr Comp Physiol* 2000, **279:**R1872-1879.

48. Jaakkola P, Mole DR, Tian YM, Wilson MI, Gielbert J, Gaskell SJ, von Kriegsheim A, Hebestreit HF, Mukherji M, Schofield CJ, et al: **Targeting of HIF-alpha to the von Hippel-Lindau ubiquitylation complex by O2-regulated prolyl hydroxylation.** *Science* 2001, **292:**468-472.

49. Daneshrad Z, Novel-Chate V, Birot O, Serrurier B, Sanchez H, Bigard AX, Rossi A: **Diet restriction plays an important role in the alterations of heart mitochondrial function following exposure of young rats to chronic hypoxia.** *Pflugers Arch* 2001, **442:**12-18.

50. Lin J, Wu H, Tarr PT, Zhang CY, Wu Z, Boss O, Michael LF, Puigserver P, Isotani E, Olson EN, et al: **Transcriptional co-activator PGC-1 alpha drives the formation of slow-twitch muscle fibres.** *Nature* 2002, **418:**797-801.

51. McClelland GB, Brooks GA: **Changes in MCT 1, MCT 4, and LDH expression are tissue specific in rats after long-term hypobaric hypoxia.** *J Appl Physiol (1985)* 2002, **92:**1573-1584.

52. Kubasiak LA, Hernandez OM, Bishopric NH, Webster KA: **Hypoxia and acidosis activate cardiac myocyte death through the Bcl-2 family protein BNIP3.** *Proc Natl Acad Sci U S A* 2002, **99:**12825-12830.

53. Kanatous SB, Davis RW, Watson R, Polasek L, Williams TM, Mathieu-Costello O: **Aerobic capacities in the skeletal muscles of Weddell seals: key to longer dive durations?** *J Exp Biol* 2002, **205:**3601-3608.

54. Howald H, Hoppeler H: **Performing at extreme altitude: muscle cellular and subcellular adaptations.** *Eur J Appl Physiol* 2003, **90:**360-364.

55. Pyner S, Coney A, Marshall JM: **The role of free radicals in the muscle vasodilatation of systemic hypoxia in the rat.** *Exp Physiol* 2003, **88:**733-740.

56. Juel C, Lundby C, Sander M, Calbet JA, Hall G: **Human skeletal muscle and erythrocyte proteins involved in acid-base homeostasis: adaptations to chronic hypoxia.** *J Physiol* 2003, **548:**639-648.

57. Stavinoha MA, RaySpellicy JW, Essop MF, Graveleau C, Abel ED, Hart-Sailors ML, Mersmann HJ, Bray MS, Young ME: **Evidence for mitochondrial thioesterase 1 as a peroxisome proliferator-activated receptor-alpha-regulated gene in cardiac and skeletal muscle.** *Am J Physiol Endocrinol Metab* 2004, **287:**E888-895.

58. Ou LC, Leiter JC: **Effects of exposure to a simulated altitude of 5500 m on energy metabolic pathways in rats.** *Respir Physiol Neurobiol* 2004, **141:**59-71.

59. Lundby C, Pilegaard H, Andersen JL, van Hall G, Sander M, Calbet JA: **Acclimatization to 4100 m does not change capillary density or mRNA expression of potential angiogenesis regulatory factors in human skeletal muscle.** *J Exp Biol* 2004, **207:**3865-3871.

60. Mason SD, Howlett RA, Kim MJ, Olfert IM, Hogan MC, McNulty W, Hickey RP, Wagner PD, Kahn CR, Giordano FJ, Johnson RS: **Loss of skeletal muscle HIF-1alpha results in altered exercise endurance.** *PLoS Biol* 2004, **2:**e288.

61. Gelfi C, Vasso M, Cerretelli P: **Diversity of human skeletal muscle in health and disease: contribution of proteomics.** *J Proteomics*, **74:**774-795.

62. Magalhaes J, Ascensao A, Soares JM, Ferreira R, Neuparth MJ, Marques F, Duarte JA: **Acute and severe hypobaric hypoxia increases oxidative stress and impairs mitochondrial function in mouse skeletal muscle.** *J Appl Physiol (1985)* 2005, **99:**1247-1253.

63. Ponsot E, Dufour SP, Zoll J, Doutrelau S, N'Guessan B, Geny B, Hoppeler H, Lampert E, Mettauer B, Ventura-Clapier R, Richard R: **Exercise training in normobaric hypoxia in endurance runners. II. Improvement of mitochondrial properties in skeletal muscle.** *J Appl Physiol (1985)* 2006, **100:**1249-1257.

64. Navet R, Mouithys-Mickalad A, Douette P, Sluse-Goffart CM, Jarmuszkiewicz W, Sluse FE: **Proton leak induced by reactive oxygen species produced during in vitro anoxia/reoxygenation in rat skeletal muscle mitochondria.** *J Bioenerg Biomembr* 2006, **38:**23-32.

65. Ripamonti M, Vigano A, Moriggi M, Milano G, von Segesser LK, Samaja M, Gelfi C: **Cytochrome c oxidase expression in chronic and intermittent hypoxia rat gastrocnemius muscle quantitated by CE.** *Electrophoresis* 2006, **27:**3897-3903.

66. Lee WJ, Kim M, Park HS, Kim HS, Jeon MJ, Oh KS, Koh EH, Won JC, Kim MS, Oh GT, et al: **AMPK activation increases fatty acid oxidation in skeletal muscle by activating PPARalpha and PGC-1.** *Biochem Biophys Res Commun* 2006, **340:**291-295.

67. Martinez ML, Landry C, Boehm R, Manning S, Cheek AO, Rees BB: **Effects of long-term hypoxia on enzymes of carbohydrate metabolism in the Gulf killifish, Fundulus grandis.** *J Exp Biol* 2006, **209:**3851-3861.

68. Fukuda R, Zhang H, Kim JW, Shimoda L, Dang CV, Semenza GL: **HIF-1 regulates cytochrome oxidase subunits to optimize efficiency of respiration in hypoxic cells.** *Cell* 2007, **129:**111-122.

69. Roels B, Thomas C, Bentley DJ, Mercier J, Hayot M, Millet G: **Effects of intermittent hypoxic training on amino and fatty acid oxidative combustion in human permeabilized muscle fibers.** *J Appl Physiol (1985)* 2007, **102:**79-86.

70. De Palma S, Ripamonti M, Vigano A, Moriggi M, Capitanio D, Samaja M, Milano G, Cerretelli P, Wait R, Gelfi C: **Metabolic modulation induced by chronic hypoxia in rats using a comparative proteomic analysis of skeletal muscle tissue.** *J Proteome Res* 2007, **6:**1974-1984.

71. Bakkman L, Sahlin K, Holmberg HC, Tonkonogi M: **Quantitative and qualitative adaptation of human skeletal muscle mitochondria to hypoxic compared with normoxic training at the same relative work rate.** *Acta Physiol (Oxf)* 2007, **190:**243-251.

72. Magalhaes J, Ferreira R, Neuparth MJ, Oliveira PJ, Marques F, Ascensao A: **Vitamin E prevents hypobaric hypoxia-induced mitochondrial dysfunction in skeletal muscle.** *Clin Sci (Lond)* 2007, **113:**459-466.

73. Haseler LJ, Lin A, Hoff J, Richardson RS: **Oxygen availability and PCr recovery rate in untrained human calf muscle: evidence of metabolic limitation in normoxia.** *Am J Physiol Regul Integr Comp Physiol* 2007, **293:**R2046-2051.

74. Vigano A, Ripamonti M, De Palma S, Capitanio D, Vasso M, Wait R, Lundby C, Cerretelli P, Gelfi C: **Proteins modulation in human skeletal muscle in the early phase of adaptation to hypobaric hypoxia.** *Proteomics* 2008, **8:**4668-4679.

75. Galbes O, Goret L, Caillaud C, Mercier J, Obert P, Candau R, Py G: **Combined effects of hypoxia and endurance training on lipid metabolism in rat skeletal muscle.** *Acta Physiol (Oxf)* 2008, **193:**163-173.

76. Aragones J, Schneider M, Van Geyte K, Fraisl P, Dresselaers T, Mazzone M, Dirkx R, Zacchigna S, Lemieux H, Jeoung NH, et al: **Deficiency or inhibition of oxygen sensor Phd1 induces hypoxia tolerance by reprogramming basal metabolism.** *Nat Genet* 2008, **40:**170-180.

77. Zhang H, Bosch-Marce M, Shimoda LA, Tan YS, Baek JH, Wesley JB, Gonzalez FJ, Semenza GL: **Mitochondrial autophagy is an HIF-1-dependent adaptive metabolic response to hypoxia.** *J Biol Chem* 2008, **283:**10892-10903.

78. Esteva S, Panisello P, Casas M, Torrella JR, Pages T, Viscor G: **Morphofunctional responses to anaemia in rat skeletal muscle.** *J Anat* 2008, **212:**836-844.

79. Lu Z, Sack MN: **ATF-1 is a hypoxia-responsive transcriptional activator of skeletal muscle mitochondrial-uncoupling protein 3.** *J Biol Chem* 2008, **283:**23410-23418.

80. Dash RK, Li Y, Kim J, Beard DA, Saidel GM, Cabrera ME: **Metabolic dynamics in skeletal muscle during acute reduction in blood flow and oxygen supply to mitochondria: in-silico studies using a multi-scale, top-down integrated model.** *PLoS One* 2008, **3:**e3168.

81. Bo H, Wang YH, Li HY, Zhao J, Zhang HY, Tong CQ: **Endurance training attenuates the bioenergetics alterations of rat skeletal muscle mitochondria submitted to acute hypoxia: role of ROS and UCP3.** *Sheng Li Xue Bao* 2008, **60:**767-776.

82. Mizuno M, Savard GK, Areskog NH, Lundby C, Saltin B: **Skeletal muscle adaptations to prolonged exposure to extreme altitude: a role of physical activity?** *High Alt Med Biol* 2008, **9:**311-317.

83. O'Hagan KA, Cocchiglia S, Zhdanov AV, Tambuwala MM, Cummins EP, Monfared M, Agbor TA, Garvey JF, Papkovsky DB, Taylor CT, Allan BB: **PGC-1alpha is coupled to HIF-1alpha-dependent gene expression by increasing mitochondrial oxygen consumption in skeletal muscle cells.** *Proc Natl Acad Sci U S A* 2009, **106:**2188-2193.

84. van Hall G, Lundby C, Araoz M, Calbet JA, Sander M, Saltin B: **The lactate paradox revisited in lowlanders during acclimatization to 4100 m and in high-altitude natives.** *J Physiol* 2009, **587:**1117-1129.

85. Chan SY, Zhang YY, Hemann C, Mahoney CE, Zweier JL, Loscalzo J: **MicroRNA-210 controls mitochondrial metabolism during hypoxia by repressing the iron-sulfur cluster assembly proteins ISCU1/2.** *Cell Metab* 2009, **10:**273-284.

86. Dutta A, Vats P, Singh VK, Sharma YK, Singh SN, Singh SB: **Impairment of mitochondrial beta-oxidation in rats under cold-hypoxic environment.** *Int J Biometeorol* 2009, **53:**397-407.

87. Scott GR, Egginton S, Richards JG, Milsom WK: **Evolution of muscle phenotype for extreme high altitude flight in the bar-headed goose.** *Proc Biol Sci* 2009, **276:**3645-3653.

88. Straadt IK, Young JF, Petersen BO, Duus JO, Gregersen N, Bross P, Oksbjerg N, Bertram HC: **Metabolic profiling of heat or anoxic stress in mouse C2C12 myotubes using multinuclear magnetic resonance spectroscopy.** *Metabolism*, **59:**814-823.

89. Wust RC, Jaspers RT, van Heijst AF, Hopman MT, Hoofd LJ, van der Laarse WJ, Degens H: **Region-specific adaptations in determinants of rat skeletal muscle oxygenation to chronic hypoxia.** *Am J Physiol Heart Circ Physiol* 2009, **297:**H364-374.

90. Gamboa JL, Andrade FH: **Mitochondrial content and distribution changes specific to mouse diaphragm after chronic normobaric hypoxia.** *Am J Physiol Regul Integr Comp Physiol*, **298:**R575-583.

91. Ponsot E, Dufour SP, Doutreleau S, Lonsdorfer-Wolf E, Lampert E, Piquard F, Geny B, Mettauer B, Ventura-Clapier R, Richard R: **Impairment of maximal aerobic power with moderate hypoxia in endurance athletes: do skeletal muscle mitochondria play a role?** *Am J Physiol Regul Integr Comp Physiol*, **298:**R558-566.

92. Schroeder JL, Luger-Hamer M, Pursley R, Pohida T, Chefd'hotel C, Kellman P, Balaban RS: **Short communication: Subcellular motion compensation for minimally invasive microscopy, in vivo: evidence for oxygen gradients in resting muscle.** *Circ Res*, **106:**1129-1133.

93. Schmutz S, Dapp C, Wittwer M, Durieux AC, Mueller M, Weinstein F, Vogt M, Hoppeler H, Fluck M: **A hypoxia complement differentiates the muscle response to endurance exercise.** *Exp Physiol*, **95:**723-735.

94. Saxena S, Shukla D, Khan YA, Singh M, Bansal A, Sairam M, Jain SK: **Hypoxia preconditioning by cobalt chloride enhances endurance performance and protects skeletal muscles from exercise-induced oxidative damage in rats.** *Acta Physiol (Oxf)*, **200:**249-263.

95. Beaudry JL, McClelland GB: **Thermogenesis in CD-1 mice after combined chronic hypoxia and cold acclimation.** *Comp Biochem Physiol B Biochem Mol Biol*, **157:**301-309.

96. Dasika SK, Kinsey ST, Locke BR: **Reaction-diffusion constraints in living tissue: effectiveness factors in skeletal muscle design.** *Biotechnol Bioeng*, **108:**104-115.

97. Li J, Zhang YB: **[Effects of different hypoxic training modes on activities of mitochondrial antioxidants and respiratory chain complex in skeletal muscle after exhaustive running in rat].** *Sheng Li Xue Bao*, **63:**55-61.

98. Pesta D, Hoppel F, Macek C, Messner H, Faulhaber M, Kobel C, Parson W, Burtscher M, Schocke M, Gnaiger E: **Similar qualitative and quantitative changes of mitochondrial respiration following strength and endurance training in normoxia and hypoxia in sedentary humans.** *Am J Physiol Regul Integr Comp Physiol*, **301:**R1078-1087.

99. Fusco A, Santulli G, Sorriento D, Cipolletta E, Garbi C, Dorn GW, 2nd, Trimarco B, Feliciello A, Iaccarino G: **Mitochondrial localization unveils a novel role for GRK2 in organelle biogenesis.** *Cell Signal*, **24:**468-475.

100. Heinonen I, Kemppainen J, Kaskinoro K, Peltonen JE, Sipila HT, Nuutila P, Knuuti J, Boushel R, Kalliokoski KK: **Effects of adenosine, exercise, and moderate acute hypoxia on energy substrate utilization of human skeletal muscle.** *Am J Physiol Regul Integr Comp Physiol*, **302:**R385-390.

101. Gamboa JL, Andrade FH: **Muscle endurance and mitochondrial function after chronic normobaric hypoxia: contrast of respiratory and limb muscles.** *Pflugers Arch*, **463:**327-338.

102. Levett DZ, Radford EJ, Menassa DA, Graber EF, Morash AJ, Hoppeler H, Clarke K, Martin DS, Ferguson-Smith AC, Montgomery HE, et al: **Acclimatization of skeletal muscle mitochondria to high-altitude hypoxia during an ascent of Everest.** *FASEB J*, **26:**1431-1441.

103. Lin YQ, Xu YO, Yue Y, Jin SY, Qu Y, Dong F, Li YP, Zheng YC: **Differences in mitochondrial gene expression profiles, enzyme activities and myosin heavy chain types in yak versus bovine skeletal muscles.** *Genet Mol Res*, **11:**2871-2877.

104. Robach P, Siebenmann C, Jacobs RA, Rasmussen P, Nordsborg N, Pesta D, Gnaiger E, Diaz V, Christ A, Fiedler J, et al: **The role of haemoglobin mass on VO(2)max following normobaric 'live high-train low' in endurance-trained athletes.** *Br J Sports Med*, **46:**822-827.

105. Chen J, Gao Y, Liao W, Huang J, Gao W: **Hypoxia affects mitochondrial protein expression in rat skeletal muscle.** *OMICS*, **16:**98-104.

106. Jacobs RA, Boushel R, Wright-Paradis C, Calbet JA, Robach P, Gnaiger E, Lundby C: **Mitochondrial function in human skeletal muscle following high-altitude exposure.** *Exp Physiol*, **98:**245-255.

107. Jacobs RA, Siebenmann C, Hug M, Toigo M, Meinild AK, Lundby C: **Twenty-eight days at 3454-m altitude diminishes respiratory capacity but enhances efficiency in human skeletal muscle mitochondria.** *FASEB J*, **26:**5192-5200.

108. Chaillou T, Koulmann N, Meunier A, Malgoyre A, Serrurier B, Beaudry M, Bigard X: **Effect of hypoxia exposure on the phenotypic adaptation in remodelling skeletal muscle submitted to functional overload.** *Acta Physiol (Oxf)*, **209:**272-282.

109. Wang D, Wei L, Wei D, Rao X, Qi X, Wang X, Ma B: **Testis-specific lactate dehydrogenase is expressed in somatic tissues of plateau pikas.** *FEBS Open Bio*, **3:**118-123.

110. Gomes AP, Price NL, Ling AJ, Moslehi JJ, Montgomery MK, Rajman L, White JP, Teodoro JS, Wrann CD, Hubbard BP, et al: **Declining NAD(+) induces a pseudohypoxic state disrupting nuclear-mitochondrial communication during aging.** *Cell*, **155:**1624-1638.

111. Morash AJ, Kotwica AO, Murray AJ: **Tissue-specific changes in fatty acid oxidation in hypoxic heart and skeletal muscle.** *Am J Physiol Regul Integr Comp Physiol*, **305:**R534-541.

112. Dromparis P, Sutendra G, Paulin R, Proctor S, Michelakis ED, McMurtry MS: **Pioglitazone inhibits HIF-1alpha-dependent angiogenesis in rats by paracrine and direct effects on endothelial cells.** *J Mol Med (Berl)*.

113. Thom R, Rowe GC, Jang C, Safdar A, Arany Z: **Hypoxic Induction of Vascular Endothelial Growth Factor (VEGF) and Angiogenesis in Muscle by Truncated Peroxisome Proliferator-Activated Receptor Gamma Coactivator (PGC)-1alpha.** *J Biol Chem*.
